# Intercellular communication in the fern endosymbiotic cyanobacterium *Nostoc azollae*

**DOI:** 10.1128/mbio.01187-25

**Published:** 2025-08-18

**Authors:** Cristina Sarasa-Buisán, Mercedes Nieves-Morión, Peter Lindblad, Sandra Nierzwicki-Bauer, Henriette Schluepmann, Enrique Flores

**Affiliations:** 1 Instituto de Bioquímica Vegetal y Fotosíntesis, CSIC and Universidad de Sevilla 16778, Seville, Spain; 2 Microbial Chemistry, Department of Chemistry-Ångström, Uppsala University 8097, Uppsala, Sweden; 3 Darrin Fresh Water Institute, Rensselaer Polytechnic Institute 8024, Troy, New York, USA; 4 Biology Department, Utrecht University 8125, Utrecht, the Netherlands; Harvard University, Cambridge, Massachusetts, USA

**Keywords:** *Azolla*, cyanobacteria, intercellular communication, *Nostoc azollae*, septal junctions, symbiosis

## Abstract

The water fern *Azolla* spp. harbors as an endobiont the N_2_-fixing, filamentous, heterocyst-forming cyanobacterium *Nostoc azollae. N. azollae* provides the fern with fixed nitrogen permitting its growth in nitrogen-poor environments. In the diazotrophic filaments of heterocyst-forming cyanobacteria, intercellular molecular exchange occurs in which heterocysts provide vegetative cells with fixed nitrogen and vegetative cells provide heterocysts with reduced carbon. Intercellular molecular exchange takes place by diffusion through septal junctions and can be probed by fluorescence recovery after photobleaching (FRAP) analysis with fluorescent markers such as calcein and 5-carboxyfluorescein. The septal junctions traverse the septal peptidoglycan (PG) through nanopores that can be visualized in isolated septal PG disks by electron microscopy. Here, we obtained from *Azolla* plants material containing the symbiotic cyanobacterium in a viable state and with different morphologies, including heterocyst-containing filaments. FRAP analysis showed effective transfer of the fluorescent markers between vegetative cells, as well as from vegetative cells to heterocysts. Interestingly, communicating and noncommunicating vegetative cells and heterocysts could be distinguished, showing conservation in the endobiont of a mechanism regulating the septal junctions. PG sacculi were also isolated and showed septal disks with arrays of nanopores that conform to those visualized in other heterocyst-forming cyanobacteria. However, a wider range of septal disk size was observed in *N. azollae*. In spite of its eroded genome, *N. azollae* maintains the intercellular communication system that is key for its growth as a multicellular organism. Additionally, labeling with the fluorescent sucrose analog esculin suggests sucrose as a source of reduced carbon for the endobiont.

**IMPORTANCE:**

The water fern *Azolla* constitutes a unique symbiotic system in which cyanobacterial endobionts capable of fixing atmospheric nitrogen provide the plant with the nitrogen needed for growth. This symbiosis is an important fertilizer for rice crops worldwide, thereby reducing the reliance on fossil fuel-derived nitrogen fertilizers. The symbiotic cyanobacterium, *Nostoc azollae*, is a heterocyst-forming strain in which a filament of cells is the organismic unit of growth. Here, we show that the intercellular molecular exchange function necessary for the multicellular behavior of the organism is conserved in the endobiotic *N. azollae*.

## INTRODUCTION

The water fern *Azolla* spp. is characterized by harboring as an endobiont the N_2_-fixing, filamentous, heterocyst-forming cyanobacterium *Nostoc azollae* (1, 2). The heterocysts are cells specialized for the fixation of atmospheric N_2_, and the endobiont allows the growth of the fern in the absence of any source of combined nitrogen (3). Cyanobacteria are characterized by performing oxygenic photosynthesis, and the fern additionally contains heterotrophic bacteria whose function in the symbiosis remains to be fully elucidated (4). *N. azollae* is transmitted vertically and contains an eroded genome of about 5.49 Mbp (5). *N. azollae* within the fern exhibits extensive morphological diversity, including vegetative filaments (with or without heterocysts), hormogonia (which lack heterocysts), and akinetes (spore-like cells).

The developmental cycles of the fern and its endobiont are tightly synchronized. *N. azollae* originates from the apical meristems of *Azolla* in which it forms the *Shoot Apical Nostoc Colony*, where small-celled, likely motile, filaments without heterocysts (hormogonia) seem to be initially present (6). As the fronds grow, *N. azollae* colonizes the leaf pocket of developing leaves that emerge just behind the meristematic tip, where *N. azollae* starts to form heterocysts. The frequency of heterocysts in filaments increases along with the leaf age, reaching frequencies of 20–30%, which is higher than that found in free-living *Nostoc* or *Anabaena* spp (7, 8). Moreover, behind the meristematic tip, the megasporocarp is formed and colonized by symbiotic filaments. In megasporocarps, endobiont cells differentiate into akinetes that later germinate as new *Azolla* sporelings emerge, colonizing newly developed apical meristems (6).

Fern-associated nitrogenase activity has been reported (3) and, actually, leaf nitrogen fixation rates respond to a developmental gradient, where mature leaves have higher heterocyst frequencies and N_2_ fixation rates (9, 10). However, after the 20th leaf of the frond, the heterocysts begin to senesce and no longer show increased N_2_ fixation (7, 11). The nitrogenase enzyme component Fe-protein (NifH) has been detected by immunogold localization in the endobiont (12). Additionally, cyanobacterial filaments isolated from the fern have been used to study transcript levels of key genes related to carbon and nitrogen assimilation, and transcripts of *nifHDK* encoding nitrogenase were indeed detected (13). Isolated filaments were also used in studies on [^13^N]N_2_ fixation showing that *N. azollae* (then called *Anabaena*) accumulates more ammonium ions than free-living cyanobacteria, suggesting that ammonium is the N-containing compound transferred from the endobiont to the fern (14).

In heterocyst-forming cyanobacteria, intercellular molecular exchange is necessary for mutual nutrition between CO_2_-fixing vegetative cells and N_2_-fixing heterocysts to allow growth (15). Intercellular communication in filamentous cyanobacteria can be studied by fluorescence recovery after photobleaching (FRAP) analysis (16). Two commonly used fluorescent tracers are calcein (622.5 Da) and 5-carboxyfluorescein (5-CF; 376.3 Da), both of which are negatively charged (17). Intercellular transfer takes place by simple diffusion (18) through proteinaceous structures known as septal junctions (19, 20). Cyanobacteria are diderm bacteria, and in filamentous cyanobacteria, the outer membrane does not enter the septum between cells, whereas each cell is surrounded by its cytoplasmic membrane and peptidoglycan (PG) (21). Septal junctions traverse the septal PG by holes termed nanopores that can be readily seen in isolated murein (PG) sacculi (22, 23).

Because, as stated above, the cyanobacterial endobionts of *Azolla* spp. are heterocyst-forming strains, we were interested in studying their multicellular properties. Here, we show effective labeling of *N. azollae* filaments freshly isolated from *Azolla filiculoides* with fluorescent markers including calcein, 5-CF, and the sucrose analog esculin. Additionally, we show intercellular exchange of fluorescent markers and the presence of septal PG nanopores in the isolated *N. azollae* filaments.

## RESULTS AND DISCUSSION

### *Azolla* juice contains metabolically active *N. azollae* cells

**Fig 1:**
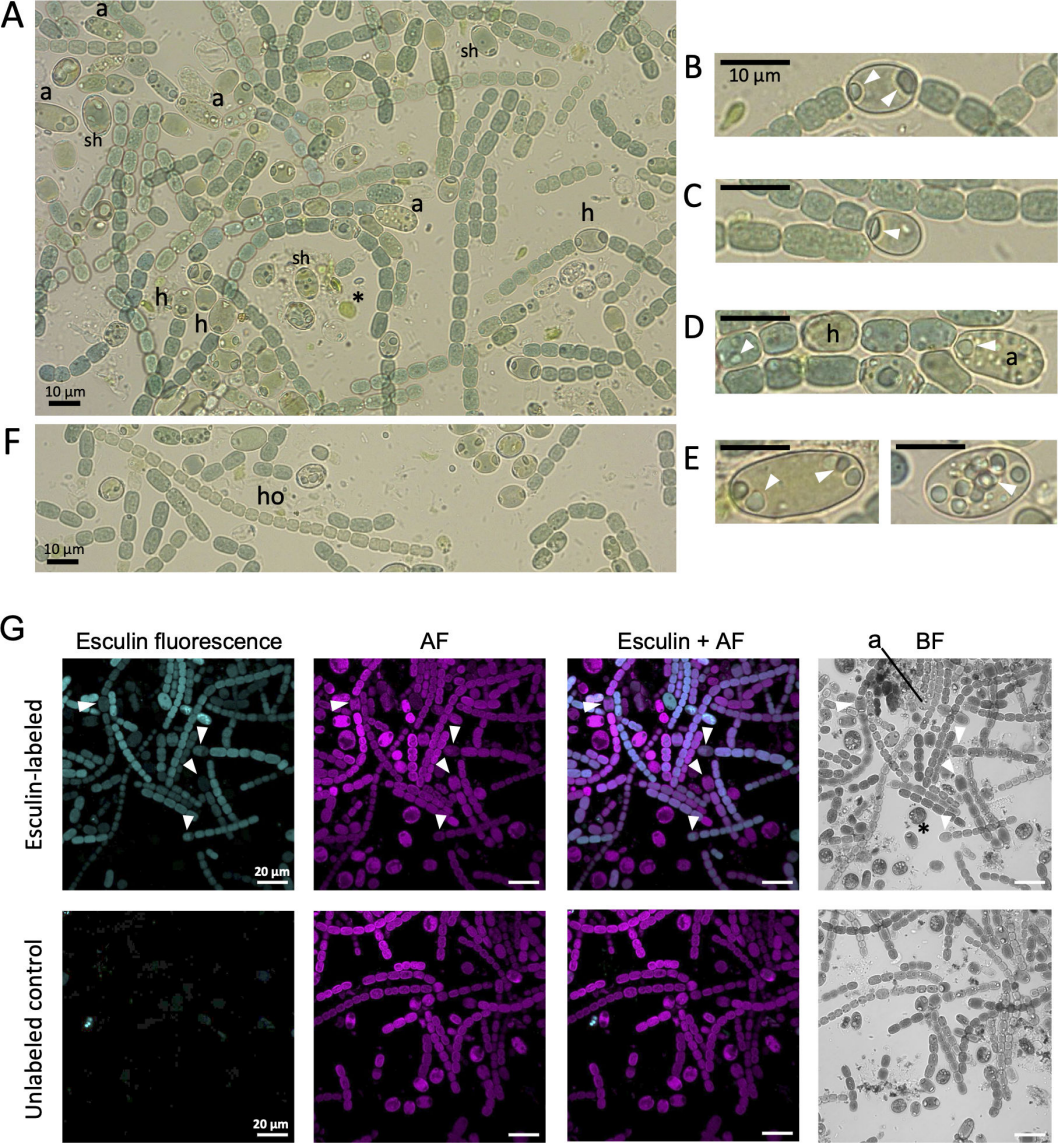
*Nostoc azollae*-enriched *Azolla* juice contains metabolically active *N. azollae* cells. (**A**) Brightfield micrograph of recently isolated *N. azollae* from *Azolla* juice depicting the variability in morphology and cell differentiation of the cyanobacterium, with the presence of heterocysts (h) and akinetes (a) at various stages of formation. Cyanophycin granules are prominent, especially at the poles of heterocysts. Note the presence of single heterocysts (sh), which are likely derived from mechanical fragmentation of filaments. Eukaryotic algae (*), which were likely present on the surface of the fern, and bacteria are also present. (**B–E**) Close-up of heterocysts and akinetes showing cyanophycin granules (some indicated by white arrowheads): (**B**) intermediate heterocyst; (**C**) terminal heterocyst; (**D**) filament with an intermediate heterocyst (h) and an akinete in formation (a), with granules visible in vegetative cells; (**E**) two akinetes, one with polar granules (left) and one with granules distributed throughout (right). (**F**) Example of a hormogonium-like filament (ho), with narrowed cells and no heterocysts. (**G**) Uptake of esculin by *N. azollae* filaments in leaf juice. (*Top row*) Esculin-labeled cells, with some heterocysts indicated by white arrowheads; a, akinete; *, eukaryotic algae. (*Bottom row*) Control with unlabeled cells. Esculin fluorescence was induced at 405 nm and acquired between 443 and 490 nm; AF, autofluorescence; BF, bright field. Scale bars for (**A–F**) are 10 µm and for (**G**), 20 µm.

Material from *A. filiculoides* referred to as “*Azolla* juice” was isolated as described in Materials and Methods (see also Fig. S1) and found to be enriched in *N. azollae* (Fig. 1). The wide variety of *N. azollae* cells and filaments observed likely reflects the fact that, using this extraction method, the isolated cyanobiont originates from all the different tissues of the plant. Therefore, we observed *N. azollae* cells in different developmental stages, that is, they can be akinetes, filaments of vegetative cells with or without heterocysts, or “hormogonia-like” filaments with narrowed cells (6), although without evident motility under our test conditions. Additionally, the juice contained bacteria that are part of the symbiosis (24), and eukaryotic algae that may be present on the surface of the fern.

To study the metabolic state of the *N. azollae* cells in the juice, the filaments were incubated with esculin, a fluorescent sucrose analog that is actively taken up into the cyanobacterial cells by ABC glucoside transporters (17, 25). Vegetative cells, heterocysts, and some akinetes were labeled well with esculin (Fig. 1G). Although hormogonium-like filaments were not specifically tracked in this study, a few small-celled filaments, similar to those described as hormogonia-like filaments (6), were observed to be labeled. However, due to the *N. azollae* isolation protocol used, which is based on homogenization of entire *Azolla* fronds rather than apex-enriched tissue, the presence of hormogonia in the preparation might be limited, as these filaments are usually more abundant in the apical regions of the fern (6). Because ABC transporters require ATP, labeling with esculin indicates that the cells were active. In contrast, the eukaryotic algae observed in the samples were not labeled, likely reflecting lack of appropriate membrane transporters.

BlastP analyses revealed that the genome of *N. azollae* encodes orthologs of some of the components of ABC glucoside transporters known to move esculin into the cells of the model heterocyst-forming cyanobacterium *Anabaena* sp. strain PCC 7120 (25, 26): GlsC and GlsD (nucleotide-binding domain [NBD] proteins), and GlsP and GlsQ (transmembrane domain [TMD] proteins) (Table 1). The *N. azollae* genome also encodes a periplasmic substrate-binding protein (SBP) similar to All1027 of *Anabaena* (26) that is homologous to that of ABC disaccharide transporters. Moreover, from the two sucrose-splitting invertases described to be involved in sucrose catabolism in heterocyst-forming cyanobacteria (27, 28), *N. azollae* conserves one that is most similar to InvB, which is known to be expressed mainly in heterocysts (27, 28) (Table 1). Labeling with the sucrose analog esculin and the presence of a putative sucrose transporter and of an invertase suggest that *N. azollae* is supplied with sucrose by the host fern. Indeed, sucrose is the main photosynthetic product in *Azolla* (29). Sugar transfer from the host must be important to support N_2_ fixation in the *N. azollae* filaments, which contain a high percentage of heterocysts, that is, a relatively low percentage of CO_2_-fixing vegetative cells (7, 8).

**TABLE 1:** Identification of potential orthologues in the *N. azollae* genome encoding proteins involved (top) in glucoside transport and sucrose metabolism and (bottom) in the formation of septal junctions and the nanopore array^*a*^

Protein name		ORF PCC 7120	Length (aa)	Reference	Potential orthologue in *N. azollae*	Length (aa)	Query cover	Identity
*Glucoside ABC transporter proteins and sucrose metabolism*								
GlsC (NBD)		*alr4781*	432	(25)	AAZO_RS14955	427	99%	77.47%
GlsP (NBD)		*all0261*	276	(25)	AAZO_RS16245	277	96%	37.74%
GlsD (TMD)		*all1823*	366	(26)	AAZO_RS14955	427	99%	42.82%
GlsQ (TMD)		*alr2532*	301	(26)	AAZO_RS23735	310	93%	35.42%
GlsR (SBP)		*all1916*	418	(26)	*			
All1027 (SBP)		*all1027*	432	(26)	AAZO_RS01530		100%	82.18%
Alr2722 (SBP)		*alr2722*	432	(26)	**		94%	22.20%
Alr4277 (SBP)		*alr4277*	461	(26)	*			
InvA		*alr1521*	468	(27)	**			
InvB		*alr0819*	483	(27)	AAZO_RS22440	482	98%	83.40%
*Septal junction and nanopore array formation*								
SepJ		*alr2338*	751	(30)	AAZO_RS17720	728	99%	50.33%
FraC		*alr2392*	179	(22, 31)	AAZO_RS00195	182	99%	61.24%
FraD		*alr2393*	343	(22, 31)	AAZO_RS00190	336	99%	62.65%
FraE		*alr2394*	267	(22, 31)	AAZO_RS00185	259	97%	68.73%
FraI		*alr4714*	232	(32)	AAZO_RS10910	210	94%	60.55%
HglK		*all0813*	727	(33)	AAZO_RS07385	706	100%	62.02%
SjcF1		*all1861*	269	(34)	AAZO_RS24465	197	94%	51.78%
SjdR		*alr0248*	218	(35)	*			
SepI		*alr3364*	521	(36)	AAZO_RS22910	497	89%	50.41%
SepT		*all2460*	426	(37)	AAZO_RS06650	420	99%	55.79%
SepN		*all4109*	235	(38)	AAZO_RS15255	238	99%	56.49%
AmiC1		*alr0092*	627	(39)	AAZO_RS04180	619	100%	64.42%
AmiC2		*alr0093*	627	(39)	AAZO_RS04175	631	100%	67.87%
LytM factor		*alr3353*	760	(40)	AAZO_RS21965	788	99%	50.06%
FurC		*alr0957*	149	(41)	AAZO_RS01485	145	96%	82.52%

^*a*^ Analyses were performed using BlastP with default parameters (% query coverage and % identity shown) with FASTA sequences of the indicated protein from *Anabaena* sp. PCC 7120 as the query and the *N. Azollae* genome annotation from 28 March 2024. (*) No potential orthologue found. (**) In case of InvA, the only similar protein encoded in the *N. Azollae* genome is InvB, and in case of Alr2722, the only similar protein encoded in the *N. Azollae* genome is the homologue of All1027 (SBP). A major facilitator superfamily protein, HepP, involved in glucoside uptake in *Anabaena* (25) has no clear homologue in *N. Azollae*.

### Freshly isolated *N. azollae* show intercellular communication

BlastP analysis showed that *N. azollae* conserves a number of the key proteins involved in intercellular molecular transfer described in *Anabaena*, such as SepJ (30, 42), FraCDE (22, 31), SepN (38), and SjcF1 (34), among others, although it does not conserve the SjdR protein involved in the structural definition of the intercellular septa (35) (Table 1). To test the potential performance of *N. azollae* in intercellular molecular transfer, FRAP analysis was set up with the cyanobacterial filaments in *Azolla* juice.

**Fig 2:**
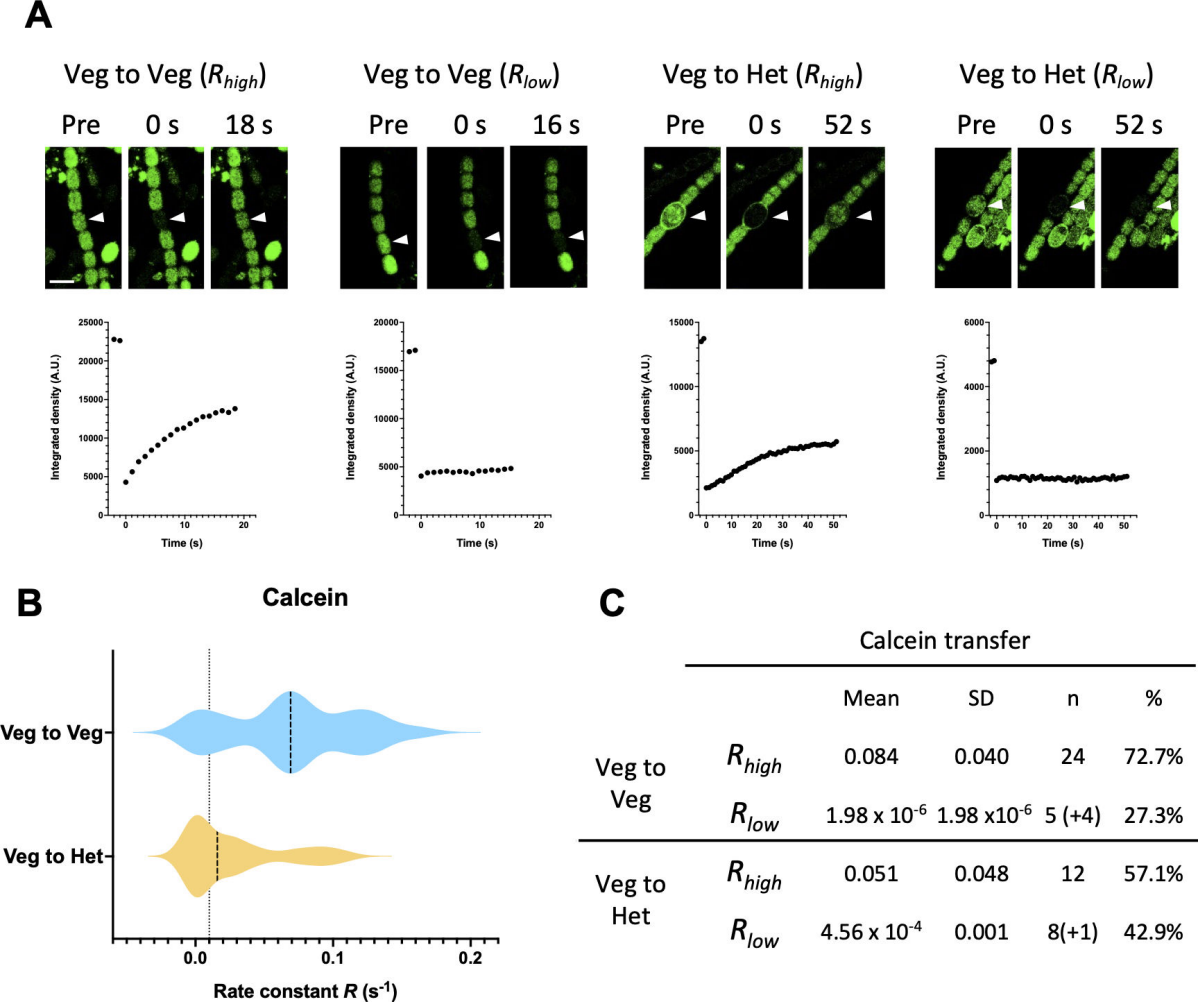
Calcein FRAP analysis of *N. azollae* filaments. (**A**) Calcein FRAP experiments. Filaments were labeled and subjected to FRAP analysis as described in Materials and Methods. The cells indicated by arrowheads were photobleached, and their fluorescence was monitored continuously. Examples of images prior (pre), and at 0 s and 16–52 s after bleaching are shown in the upper panel. Scale bars, 10 µm. Non-normalized fluorescence recovery curves for their respective bleached cells are shown below (note the two points of pre-bleaching fluorescence at the left in each graph). (**B**) Violin plot of recovery rate constants (*R*) for calcein recovery between vegetative cells (Veg to Veg) and from vegetative cells to a heterocyst (Veg to Het). The dotted line indicates the threshold, *R =* 0.01 s^−1^, that is used to discriminate two populations of cells, communicating cells (*R_high_, R* ≥ 0.01 s^−1^) and noncommunicating cells (*R_low_*, *R <* 0.01 s^−1^). Dashed lines depict the median values. (**C**) Table summarizing the mean recovery rate constants (*R*, s^−1^), grouped by communicating (*R_high_*) and noncommunicating (*R_low_*) cells observed for calcein transfer. Numbers in parentheses represent noncommunicating cells that did not adjust to the model, making it not possible to obtain an *R* number, but are included to calculate a percentage.

**Fig 3:**
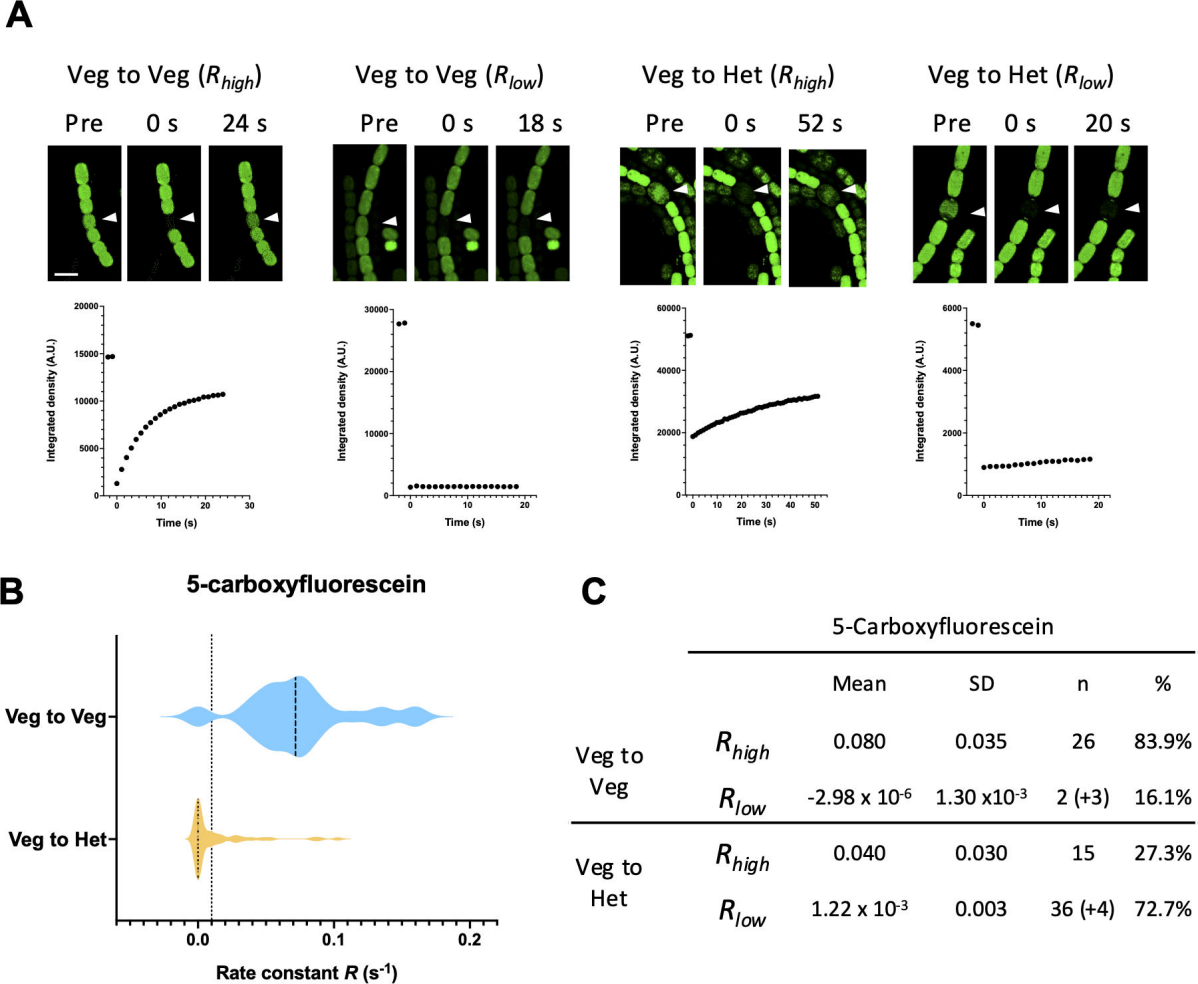
5-Carboxyfluorescein FRAP analysis of *N. azollae* filaments. (**A**) 5-CF FRAP experiments. Filaments were labeled with 5-CF and subjected to FRAP analysis as described in Materials and Methods. The cells indicated by arrowheads were photobleached, and their fluorescence was monitored continuously. Examples of images prior (pre), and at 0 s and 16–52 s after bleaching are shown in the upper panel. Scale bars, 10 µm. Non-normalized fluorescence recovery curves for their respective bleached cells are shown below (note the two points of pre-bleaching fluorescence at the left in each graph). (**B**) Violin plot of recovery rate constants (*R*) for 5-CF recovery between vegetative cells (Veg to Veg) and from vegetative cells to a heterocyst (Veg to Het). The dotted line indicates the threshold, *R =* 0.01 s^−1^, that is used to discriminate two populations of cells, communicating cells (*R_high_, R* ≥ 0.01 s^−1^) and noncommunicating cells (*R_low_*, *R <* 0.01 s^−1^). Dashed lines depict the median values. (**C**) Table depicting the mean recovery rate constants (*R*, s^−1^), grouped by communicating cells (*R_high_*) and noncommunicating cells (*R_low_*) observed for 5-CF transfer. Numbers in parentheses represent the noncommunicating cells that did not adjust to the model, making it not possible to obtain an *R* number, but are included to calculate a percentage.

Calcein AM and 5-CF AM are hydrophobic compounds that enter the cells by diffusion through the cytoplasmic membrane. Once inside cells, these compounds are hydrolyzed by nonspecific cytoplasmic esterases and become fluorescent and hydrophilic (membrane-impermeable) probes, being reftained only by living cells, and can therefore be used as viability markers for discriminating between potential live or dead cells (43, 44). Cyanobacterial filaments in the *Azolla* juice were well labeled with calcein and 5-CF (see “Pre” in Fig. 2A and 3A), consistent with cells being alive, corroborating the results with esculin described above. These provided filaments that were suitable for FRAP analysis.

The transfer of calcein between vegetative cells took place in about 73% of the cells tested, with a recovery rate constant, *R*, of about 0.084 s^−1^ (Fig. 2B and C). This is similar to values described for free-living *Anabaena* grown in the presence of nitrate or incubated in the absence of a source of combined nitrogen (16, 17, 31, 45). The rest of the cells examined (about 27%) showed *R* < 0.01 s^−1^, which indicates that they were noncommunicating cells as defined previously (31). Transfer of calcein from a vegetative cell to a heterocyst was found to take place in about 57% of the heterocysts with an *R* value of about 0.051 s^−1^, which is lower than between vegetative cells as has been described for *Anabaena* (16). The rest of the heterocysts tested were noncommunicating (about 43%, *R* < 0.01 s^−1^).

We also tested 5-CF transfer between vegetative cells and from vegetative cells to heterocysts. About 84% of the vegetative cells tested were communicating with a mean *R* value of 0.08 s^−1^, which is identical to a value described for *Anabaena* (17) and about 16% of the vegetative cells were noncommunicating (Fig. 3B and C). 5-CF transfer to heterocysts took place only in about 27% of the heterocysts tested, and the observed *R* value was relatively low, 0.040 s^−1^. Transfer of 5-CF from vegetative cells to heterocysts has not been reported before for any heterocyst-forming cyanobacterium, but our results show that this marker can also be used to test molecular transfer between the different cell types (vegetative cells and heterocysts).

The high percentage of noncommunicating heterocysts for 5-CF was also noticed by the observation of non-stained heterocysts before the FRAP routine, as in several cases a heterocyst within a filament exhibited weak or no fluorescence when stained with 5-CF, in spite of being surrounded by labeled vegetative cells (Fig. S2). This result indicates a low or null permeability of the heterocyst cell wall to 5-CF AM and suggests that the heterocysts labeled received 5-CF from their adjacent vegetative cells. Because, as shown with esculin (17), heterocysts lose communication as they senesce, the relatively low number of communicating heterocysts detected with 5-CF likely reflects the heterogeneity of *N. azollae* cells in the *Azolla* juice. The percentage of communicating heterocysts detected with calcein was, however, larger than that determined with 5-CF (Fig. 2C and 3C), consistent with the idea that calcein and 5-CF transfer are not completely equivalent and different types of septal junctions may have a preference for one or the other marker (17, 42, 46, 47).

### *N. azollae* contains a rich array of septal nanopores

**Fig 4:**
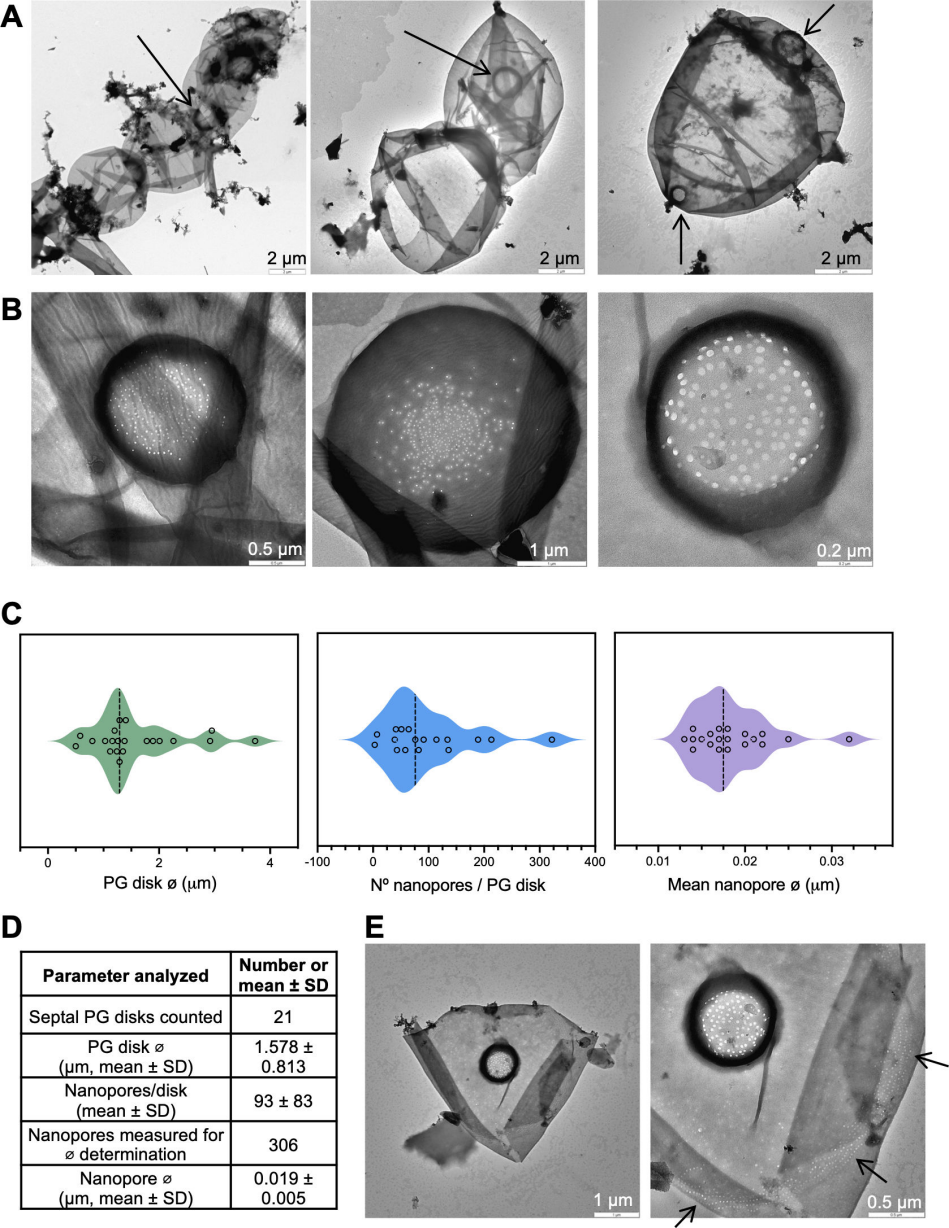
Nanopores in septal peptidoglycan disks of *N. azollae* filaments. (**A**) Murein sacculi of a fragment of a filament (*left*), of two cells (*middle*) and of a single cell (separated from other cells in the filament; *right*); the arrows point to septal PG disks. (**B**) Examples of septal disks and corresponding nanopore arrays for an average diameter disk, ⌀ 1.38 µm (*left*)*,* an over-average disk, ⌀ 3.73 µm (*middle*)*,* and a lower-than-average disk, ⌀ 0.80 µm (*right*). The PG was isolated and visualized by TEM as described in Materials and Methods. Scale bars are shown. (**C**) Violin plots depicting the measurements of PG disk diameter (µm), number of nanopores/disk, and nanopore diameters (µm). Dashed lines depict the median values. (**D**) Table summarizing the means and standard deviations of nanopore numbers per septal PG disk and diameters of PG disks and nanopores. (**E**) The sacculus harboring the PG disk shown in panel (B; *right*), here visualized at different magnifications, may correspond to a heterocyst because it contains “arcs of small pores” (arrows), which may be involved in polysaccharide export; alternatively, it may correspond to a vegetative cell sacculus holding the machinery for type IV pili (see Discussion in the text).

Murein (PG) sacculi were isolated from filaments/cells in the *Azolla* juice and prepared and visualized by transmission electron microscopy (TEM) as described in Materials and Methods. Murein sacculi corresponding to one or more cells were observed that contained septal disks (Fig. 4A). The septal PG disks had a mean diameter of about 1.6 µm, which is larger than that described for *Anabaena* (about 0.9 µm [22]), but disks clearly smaller and larger than that mean size could be observed (Fig. 4B).

All the disks observed contained nanopores, with 93 ± 83 nanopores per septal disk (mean ± SD), indicating a wide variability (Fig. 4D). In other studies, 100 to 250 nanopores (also termed “microplasmodesmata”) per septal disk were reported for *Anabaena variabilis* and *Anabaena cylindrica *(48), about 155 for *Nostoc punctiforme *(23), and about 75 (17) or 41 (31) for *Anabaena*. The mean diameter of the *N. azollae* nanopores was about 19 nm (Fig. 4C), similar to *Anabaena* (17). Because there was some variability in nanopore number and disk and nanopore diameters, we examined possible correlations between these parameters. There was not a significant correlation between nanopore number and nanopore diameter (*R*^2^ = 0.124; *P* = 0.165) nor between septal disk and nanopore size (*R*^2^ = 0.107; *P* = 0.160), whereas a moderate positive correlation was observed between nanopore number and septal disk diameter (*R*^2^ = 0.277; *P* = 0.030) (Fig. S3). Thus, larger septal disks may accommodate a somewhat larger number of nanopores.

In two cases, arcs of small pores were observed in the sacculus outside of the septal disks (one example shown in Fig. 4E). These arcs of small pores have been described in heterocyst sacculi of *Anabaena*, where they have been suggested to be involved in polysaccharide translocation across the PG (22). However, they can also be present in vegetative cells (49), in which they may hold the type IV pili machinery (50, 51). An interesting possibility is that the sacculus containing the arc of pores belongs to a hormogonial cell.

Our observation of nanopores in the septal PG disks of the endobiont is consistent with the presence of orthologues of key *Anabaena* proteins involved in intercellular molecular transfer in the *N. azollae* genome. In particular, the AmiC1 and AmiC2 amidases and an AmiC regulatory factor (LytM), which are important for the formation of nanopores in septal disks (39, 40), are conserved in *N. azollae* (Table 1).

### Concluding remarks

The results presented here show that the juice prepared from *Azolla* plants contains live *N. azollae* cells, as deduced from the observation that *N. azollae* cells freshly isolated from the fern are metabolically active. This is consistent with previous work in which freshly isolated *N. azollae* cells were used to study, for example, [^13^N]N_2_ fixation and the fate of fixed ^13^N (14). Further, labeling with the fluorescent sucrose analog esculin suggests sucrose as a source of reduced carbon that can be transferred from the host to the endobiont. Support of an N_2_-fixing, heterocyst-forming symbiont with reduced carbon from the host is also observed in the symbiotic systems *Hemiaulus-Richelia* (diatom-cyanobacterium) (52, 53), *Anthoceros-Nostoc* (bryophyte-cyanobacterium) (54, 55), *Gunnera-Nostoc* (angiosperm-cyanobacterium) (56, 57) and, likely, *Cycas-Nostoc* (gymnosperm-cyanobacterium) (58, 59), in all of which the cyanobacterium has a high percentage of heterocysts. Therefore, it can be generalized that cyanobacterial symbionts that have a relatively small number of photosynthesizing vegetative cells per heterocyst are likely to require support with reduced C from their hosts to perform nitrogen fixation at high levels.

Most of the extracted *N. azollae* was largely present as filaments, and many filaments contained heterocysts. FRAP analysis showed substantial activities of intercellular transfer of calcein and 5-CF, indicating that the cells in the *N. azollae* filaments can communicate with each other. This aligns with the presence in the eroded genome of *N. azollae* of orthologues for a majority of the key proteins involved in the functionality of the intercellular molecular transfer machinery. Intercellular communication is essential for the growth of the filaments, in which the heterocysts provide the vegetative cells with fixed N, and the vegetative cells provide heterocysts with reduced C. Additionally, noncommunicating vegetative cells were observed, indicating that the mechanism(s) that regulate septal junction opening/closing in *Anabaena *(20), with closing making a cell noncommunicating (31), are conserved in *N. azollae*. Noncommunicating heterocysts were also observed, which may correspond to senescent heterocysts as previously discussed (17). Thus, in spite of an endophytic lifestyle, *N. azollae* filaments retain a fully active intercellular communication system that allows its growth as a multicellular organism. Additionally, because of the extra cell wall layers of the heterocyst, it is possible that the exchange of nutrients (reduced C, fixed N) between fern and endobiont occurs through the vegetative cells, as is also likely the case in the *Hemiaulus hauckii-Richelia euintracellularis* symbiosis (60). This would give further importance to the intercellular molecular exchange within the cyanobacterial filament.

The range of sizes of the septal PG disks is wider in *N. azollae* than in the *Anabaena* model (22). This observation may reflect the presence in the *Azolla* juice of different cell types, including the vegetative cells and heterocysts of diazotrophic filaments and the cells of hormogonia or hormogonia-like filaments. In diazotrophic filaments, vegetative cell-vegetative cell septa and vegetative cell-heterocyst septa may have different-sized PG disks. Additionally, the hormogonial cells are smaller than those in diazotrophic filaments, likely involving smaller intercellular septa and, thus, smaller PG disks.

## MATERIALS AND METHODS

### *Azolla* fern culturing

This study used *A. filiculoides,* strain Galgenwaard (2). Adult *Azolla* sporophytes were grown in a modified Standard *Azolla* Medium “SAM,” a variation of IRRI2 medium (IRRI; International Rice Research Institute) (61), under a 16 h light and 8 h dark diel cycle at 20°C. To maintain optimal growth conditions, white light (100 µmol m⁻² s⁻¹) was supplemented with Far-Red light from an APEXstrip Bundle 730 nm – 16W system (Crescience)*.*

### *Azolla* juice extraction

*N. azollae*-enriched *Azolla* juice was obtained following the protocol visually documented in Fig. S1. Briefly, an amount of ~10 g of *Azolla* fronds was placed in dH_₂_O-rinsed ziplock bags and mixed with 10 mL of BG11_0_ medium (62), with ferric ammonium citrate substituted by ferric citrate. The juice was extracted by squeezing the ziplock bags containing the fronds using a pasta maker machine by gently crushing the plant to minimize the release of oxidants. The juice obtained from two to three extractions was gently centrifuged at 3,000 × *g* for 5 min at room temperature. The supernatant was discarded, and the resulting pellet, containing material enriched in *N. azollae,* was resuspended in half of the total extraction volume of fresh BG11_0_ medium. For nanopore analysis, pelleted cells were extracted in dH_2_O instead of BG11_0_ medium, and the pellet was stored at −20°C until PG isolation.

### Labeling with the sucrose-analogue esculin

Esculin labeling was performed with a protocol adapted from the previously described one (17). The cells from a 1 mL aliquot of the *N. azollae*-enriched *Azolla* juice suspension (obtained as described above) were harvested by gentle centrifugation at room temperature, resuspended in 500 µL of fresh BG11_0_ medium, and mixed with 15 µL of a saturated esculin hydrate solution (~5 mM, Sigma-Aldrich) in water. The mixture was then incubated in the dark for 1 h at 30°C with gentle shaking, followed by three washes with BG11_0_ medium. The cells were subsequently incubated in the dark for another 15 min in 1 mL of medium at 30°C with mild agitation. After this, the cells were washed again, concentrated about 20-fold, and applied (20–40 µL) onto BG11_0_ medium plates solidified with 1% (wt/vol) Difco Bacto agar. Pieces of agar from the BG11_0_ medium plate carrying cells/filaments were cut out and covered with a coverslip for visualization by confocal microscopy with an Olympus FLUOVIEW FV3000 confocal laser-scanning microscope equipped with a UPlanApo 60× 1.5 NA oil immersion objective. Esculin was excited at 405 nm and fluorescence was detected within the 443–490 nm range. 

### FRAP experiments

Calcein and 5-CF labeling was performed as previously reported by Mullineaux et al. (16) and Merino-Puerto et al. (46), respectively. Briefly, for calcein staining, cells from 0.5 mL of *N. azollae*-enriched *Azolla* juice suspension (obtained as described above in BG11_0_ medium) were harvested by gentle centrifugation, washed two times, resuspended in 0.5 mL of fresh BG11_0_ medium, and mixed with 10 µL of calcein-AM (1 mg/mL in dimethyl sulfoxide). The suspensions were incubated in the dark at 30°C for 90 min, and cells were then harvested and washed three times in fresh, dye-free BG11_0_ medium and resuspended in 175 µL of dye-free BG11_0_ medium. For 5-CF, 1 mL of *N. azollae*-enriched *Azolla* juice suspension was harvested, washed two times, and resuspended in 1 mL of fresh BG11_0_ medium, and then mixed with 4 µL of 5-CF diacetate AM (5 mg/mL in dimethyl sulfoxide). The suspension was incubated in the dark at 30°C for 30 min, and filaments were then harvested and washed three times in fresh, dye-free BG11_0_ medium and resuspended in 250 µL of dye-free BG11_0_ medium.

Cell suspensions from calcein and 5-CF labeling were spotted and spread onto 30 mL plates of BG11_0_-agar medium and placed in a temperature-controlled sample holder with a glass cover slip on top. All measurements were carried out at 30°C. Images were collected with an Olympus FLUOVIEW FV3000 confocal laser-scanning microscope equipped with a UPlanApo 60× 1.5 NA oil immersion objective. Excitation was achieved with a 488 nm argon laser and fluorescent emission was monitored by collection across windows of 500–520 nm. After an initial image was recorded, the bleach was carried out by a pre-set FRAP routine previously described (16) using 20% laser and occasionally 40% laser for cases when total bleach was difficult to obtain (large-sized heterocysts). In all cases, values of fluorescence recovery were normalized to the pre-bleaching value. Kinetics of transfer of calcein and 5-CF was computed with the Fiji processing package from ImageJ (63) and the recovery rate constant, *R*, was calculated as previously described (25) for both fluorescent markers.

### Nanopore analysis

The cells from *N. azollae*-enriched *Azolla* juice suspensions were obtained as described above and PG sacculi were isolated and analyzed as previously described (17, 23). The purified PG sacculi were placed on formvar/carbon film coated 150-mesh copper grids (*Electron Microscopy Sciences*) and stained with 1% (wt/vol) uranyl acetate. Samples were visualized using a Zeiss Libra 120 Plus electron microscope at 120 kV.

## SUPPLEMENTAL MATERIAL

Supplemental material mbio.01187-25-s0001.pdf Figures S1 to S3.

ASM does not own the copyrights to Supplemental Material that may be linked to, or accessed through, an article. The authors have granted ASM a non-exclusive, world-wide license to publish the Supplemental Material files. Please contact the corresponding author directly for reuse.
